# Identification of risk factors for delirium, cognitive decline, and dementia after cardiac surgery (FINDERI—find delirium risk factors): a study protocol of a prospective observational study

**DOI:** 10.1186/s12872-022-02732-4

**Published:** 2022-06-30

**Authors:** Monika Sadlonova, Jonathan Vogelgsang, Claudia Lange, Irina Günther, Adriana Wiesent, Charlotte Eberhard, Julia Ehrentraut, Mareike Kirsch, Niels Hansen, Hermann Esselmann, Charles Timäus, Thomas Asendorf, Benedict Breitling, Mohammed Chebbok, Stephanie Heinemann, Christopher Celano, Ingo Kutschka, Jens Wiltfang, Hassina Baraki, Christine A. F. von Arnim

**Affiliations:** 1 Department of Cardiovascular and Thoracic Surgery, University of Göttingen Medical Center, Robert-Koch-Street 40, 37075 Göttingen, Germany; 2 Department of Psychosomatic Medicine and Psychotherapy, University of Göttingen Medical Center, Göttingen, Germany; 3 DZHK (German Center for Cardiovascular Research), Partner Site, Göttingen, Germany; 4 Department of Psychiatry, Massachusetts General Hospital, Boston, USA; 5 Department of Psychiatry, Harvard Medical School, Boston, USA; 6 Department of Psychiatry, Translational Neuroscience Laboratory, McLean Hospital, Harvard Medical School, Boston, USA; 7 Department of Psychiatry and Psychotherapy, University of Göttingen Medical Center, Göttingen, Germany; 8 Department of Geriatrics, University of Göttingen Medical Center, Göttingen, Germany; 9 Department of Medical Statistics, University of Göttingen Medical Center, Göttingen, Germany; 10 Department of Cardiology and Pneumology, University of Göttingen Medical Center, Göttingen, Germany; 11 German Center for Neurodegenerative Diseases (DZNE), Göttingen, Germany; 12 Neurosciences and Signaling Group, Institute of Biomedicine (iBiMED), Department of Medical Sciences, University of Aveiro, Aveiro, Portugal

**Keywords:** Delirium, Cognitive decline, Dementia, Delirium risk assessment, Cardiac surgery, Biomarkers

## Abstract

**Background:**

Postoperative delirium is a common complication of cardiac surgery associated with higher morbidity, longer hospital stay, risk of cognitive decline, dementia, and mortality. Geriatric patients, patients undergoing cardiac surgery, and intensive care patients are at a high risk of developing postoperative delirium. Gold standard assessments or biomarkers to predict risk factors for delirium, cognitive decline, and dementia in patients undergoing cardiac surgery are not yet available.

**Methods:**

The FINDERI trial (FINd DElirium RIsk factors) is a prospective, single-center, observational study. In total, 500 patients aged ≥ 50 years undergoing cardiac surgery at the Department of Cardiovascular and Thoracic Surgery of the University of Göttingen Medical Center will be recruited. Our primary aim is to validate a delirium risk assessment in context of cardiac surgery. Our secondary aims are to identify specific preoperative and perioperative factors associated with delirium, cognitive decline, and accelerated dementia after cardiac surgery, and to identify blood-based biomarkers that predict the incidence of postoperative delirium, cognitive decline, or dementia in patients undergoing cardiac surgery.

**Discussion:**

This prospective, observational study might help to identify patients at high risk for delirium prior to cardiac surgery, and to identify important biological mechanisms by which cardiac surgery is associated with delirium. The predictive value of a delirium screening questionnaire in cardiac surgery might be revealed. Finally, the identification of specific blood biomarkers might help to predict delirium, cognitive decline, and dementia in patients undergoing cardiac surgery.

*Trial registration*: Ethics approval for this study was obtained from the IRB of the University of Göttingen Medical Center. The investigators registered this study in the German Clinical Trials Register (DRKS; https://www.drks.de) (DRKS00025095) on April 19th, 2021.

**Supplementary Information:**

The online version contains supplementary material available at 10.1186/s12872-022-02732-4.

## Background

Postoperative delirium (POD) is a common complication of cardiac surgery [1, 2]. Several studies have already shown that geriatric patients, patients undergoing cardiac surgery, and intensive care patients are at a high risk of developing POD [3–9]. Recent data suggest an age-dependent incidence of post-cardiac surgery delirium: 21.4% of patients aged ≥ 65 suffered from POD, and POD was observed in 33.5% of patients aged > 80 [1]. Patients with delirium are more likely to have a prolonged hospitalization, a pre-existing dementia, and to die during hospitalization [10]. For example, a meta-analysis including almost 2,939 patients showed that delirium is associated with an increased risk of death after an average follow-up of 22.7 months (hazard ratio [HR] 1.95, 95% CI 1.51–2.52), as well as an increased risk of institutionalization (OR 2.41, 95% CI 1.77–3.29) [11]. In several studies, delirium was associated with an increased risk of postoperative cognitive dysfunction and dementia [10–12]. For example, a recent study showed an association between delirium severity and subsequent 3-year cognitive impairment [13]. In patients who experienced the most severe delirium, the rate of cognitive decline nearly tripled [13].

It has recently been shown that surgery, in particular cardiac surgery, is associated with increased markers for neuronal damage and neurodegeneration, such as total-Tau and neurofilament light chain (NfL) [14–17]. These effects, including hypoxemia and disruption of the blood-brain barrier, seem to be caused by the entire procedure and not solely by inhalational anesthesia, as previously hypothesized [18]. It is likely that patients with an underlying neurodegenerative disorder, although still clinically inapparent, might experience a significant “second hit” that accelerates the neurodegenerative processes causing cognitive decline after these interventions. This hypothesis is supported by pre-surgery cerebrospinal fluid (CSF) measurements of Amyloid-β_42_ (Aβ_42_), a key biomarker for Alzheimer’s disease (AD) [19]. Furthermore, Evered et al. report decreased CSF Aβ_42_ levels in patients suffering from cognitive decline after general surgery [19].

Inflammation might also help to explain the relationships between surgery and POD, cognitive decline, and dementia. C-reactive protein (CRP) has been linked to delirium in individuals with critical illness and other populations [20]. Recently published studies showed that POD following cardiac surgery is associated with a significant increase in the inflammatory markers CC-chemokine ligand 2 (CCL2) [21], interleukin-6 (IL-6) [22], and fibroblast growth factor (FGF) 21 and 23 [23]. Several studies have analyzed the association between POD and plasma levels of glial fibrillary acidic protein (GFAP), a neuroinflammatory marker [24]. Plasma GFAP levels are known to be elevated in cases with astroglial damage [25] and neurodegenerative processes such as AD [26–30]. Therefore, GFAP might exhibit a link between inflammation and neurodegeneration.

The ability to identify patients at risk prior to surgery is essential for delirium prevention and advanced care planning [31]. In previous research, preoperative and pre-existing risk factors of delirium in patients undergoing cardiac surgery, as well as perioperative and intraoperative risk factors of delirium, were analyzed. For example, a study analyzing 1,206 patients undergoing open-heart surgery identified higher age and longer aortic cross-clamp time as important risk factors for POD [32]. Furthermore, a retrospective analysis of 1,797 patients identified eight independent factors for the development of post-cardiac surgery delirium: age, low ejection fraction, diabetes, extracardiac arteriopathy, postoperative atrial fibrillation, pneumonia, elevated creatinine, and prolonged hospitalization stay. Additionally, these independent factors are all markers for pre-interventional frailty [1]. However, these studies did not distinguish between pre-existing risk factors that can identify patients with high risk for delirium and peri- and intraoperative factors that can have the potential for modified operative approaches. From a clinical point of view, this is an important distinction.

While there are well-established predictors for delirium incidence, a standardized screening assessment for the risk of POD is not available [33–35]. Lindroth et al. [31] examined numerous assessments for delirium prediction. Overall, the American College of Surgeons (ACS) National Surgical Quality Improvement Program risk calculation for serious complication (NSQIP-SC) and the Trail Making Test B (TMTB), a measure of executive function, have been shown to predict POD severity using advanced modeling techniques [31]. However, further research is needed to establish a reliable and time-efficient screening instrument for delirium in the preoperative daily routine of cardiac surgery.

Our primary aim is to validate an existing delirium risk assessment in context of cardiac surgery. This delirium risk assessment was previously developed in the multicenter, cluster-randomized PAWEL study [36] and incorporates information from the medical record, patient report, and brief bedside cognitive testing that can be performed preoperatively. Secondary aims are i) to identify specific preoperative and perioperative factors associated with delirium, cognitive decline, and accelerated dementia after cardiac surgery, and ii) to identify blood-based biomarkers that predict the incidence of postoperative delirium, cognitive decline, or dementia in the context of cardiac surgery.

## Methods

### Study design

FINDERI is a prospective, single-center, observational study. The primary aim is to validate a delirium risk assessment in context of cardiac surgery. The secondary study aims are to identify specific pre- and perioperative risk factors and biomarkers for POD and cognitive decline in patients undergoing cardiac surgery and to investigate if cardiac surgery accelerates cognitive decline in the presence of Aβ pathology. Ethical approval for this study protocol (Version 2) was obtained from the Ethics Committee of the University of Göttingen Medical Center (#20/11/20) on February 16th, 2021. The investigators registered this study in the German Clinical Trials Register (DRKS; https://www.drks.de) (DRKS00025095) on April 19^th^, 2021.

### Study setting and participants

**Fig. 1:**
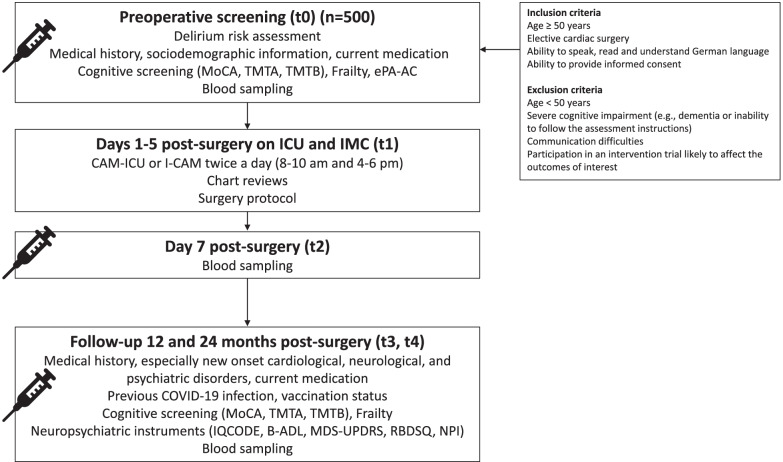
Trial flow diagram. *Note:* B-ADL: Bayer activities of daily living scale; CAM: Confusion assessment method; ICU: Intense care unit; IMC: Intermediate care unit; IQCODE: Informant questionnaire on cognitive decline in the elderly; MDS-UPDRS: Movement disorder society-unified Parkinson's disease rating scale; MoCA: Montreal cognitive assessment; NPI: Neuropsychiatric inventory; RBDSQ: REM sleep behavior disorder screening questionnaire; TMTA: Trail making test A, TMTB: Trail making test B

In total, 500 patients aged over 50 years old undergoing cardiac surgery will be recruited at the Department of Cardiovascular and Thoracic Surgery of the University Medical Center Göttingen, Germany. Inclusion and exclusion criteria are summarized in Table 1. Potentially eligible participants will be identified by preoperative schedules. Informed consent will be collected by study team after providing detailed study information and prior to baseline assessment and biomarker sampling. Initially, preoperative screening will be assessed via medical history, a screening questionnaire, and laboratory measurements for identification of risk factors for delirium, cognitive decline, and dementia (t0). Furthermore, delirium symptoms will be assessed over the first five postoperative days (t1), and biomarkers will be measured on the 7th postoperative day (t2) to address the impact on neurodegenerative and neuroinflammatory markers after cardiac surgery. We will provide two follow-up visits for all patients 12 months (t3) and 24 months (t4) after surgery (Fig. 1, Table 2).

**Table 1:** Inclusion and exclusion criteria of the FINDERI study

**Inclusion criteria**
Age ≥ 50 years
Hospitalized in the Department of Cardiovascular and Thoracic Surgery for an elective cardiac surgery
Ability to speak, read and understand German
Ability to provide informed consent
**Exclusion criteria**
Age < 50 years
Severe cognitive impairment (e.g., dementia or inability to follow the assessment instructions)
Communication difficulties (e.g., severe hearing loss, aphasia)
Participation in an intervention trial likely to affect the outcomes of interest

**Table 2:** Visit schedule and study assessments

Study assessments	t0	t1	t2	t3	t4
	Pre-surgery	Post-surgery Day 1–5	Post-surgery Day 7	Post-surgery 12 m	Post-surgery 24 m
Inclusion and exclusion criteria	X				
Clinical data and medical history	X			X	X
Delirium risk assessment modified from the PAWEL trial	X			X	X
Canadian study of health and aging (CSHA) frailty scale	X			X	X
Hand grip strength	X				
Montreal cognitive assessment (MoCA)	X			X	X
Trail making test (TMTA and TMTB)	X			X	X
Laboratory measurements	X		X	X	X
Outcome-oriented nursing assessment instrument AcuteCare 1.1 (ePA-AC)	X				
Confusion assessment method (CAM-ICU/I-CAM)		X			
Chart review		X			
Surgery protocol			X		
Informant questionnaire on cognitive decline in the elderly (IQCODE)				X	X
Bayer activities of daily living scale (B-ADL)				X	X
Movement disorder society-unified Parkinson's disease rating scale (MDS-UPDRS)				X	X
REM sleep behavior disorder screening questionnaire (RBDSQ)				X	X
Neuropsychiatric inventory (NPI)				X	X

### Preoperative screening (t0)

Upon admission, preoperative delirium risk will be assessed using a questionnaire (Additional file 1: Supplement 1) adapted from the multicenter, cluster-randomized PAWEL study [36]. This delirium risk assessment will be validated in context of cardiac surgery. This preoperative risk assessment questionnaire includes the following assessments: A) Geriatric check (mobility, statutory level of independency, cognition, psychological symptoms, and previous hospital stay); B) Short 6-item cognitive screening; C) General information including results from A) and B), age > 80, laboratory measurement (e.g., increased creatine, CRP, hemoglobin, decreased protein), and an American Society of Anesthesiologists (ASA) score ≥ 3 [37]. The patient's current medications are also noted; however, particular attention is paid to the number of medications and medications with a deliriogenic risk. Furthermore, preoperative diagnosis of depression, stroke, dementia, and previous hospital stays within the last year will be assessed. Additionally, the patient’s level of care and whether the patient is a nursing home resident will be assessed. Study participants will be asked about previous delirium and a recent increase in number of falls. Another component of the survey is the self-reported subjective memory impairment (SMI), alcohol use, and smoking status. Finally, the patient's handgrip strength will be measured by the Jamar® Hydraulic Hand Dynamometer.

Furthermore, basic sociodemographic information will be collected, including marital status, number of children, immigrant background, level of education, occupation, living arrangement, and care level. Moreover, an examination of previous cardiac and noncardiac comorbidities will be provided (Additional file 2: Supplement 2). Frailty will be assessed using the 7-point Clinical Frailty Scale of the Canadian Study of Health and Aging (CSHA Frailty Scale, 1-very fit up to 7-severely frail) [38]. In addition, the outcome-oriented nursing assessment instrument AcuteCare 1.1 (ePA-AC) [39], whereby the software generates a delirium risk score and a self-care score, will be assessed from the MEONA software. Factors biasing the adequate elicitation of the ePA-AC (Additional file 3: Supplement 3), such as the understaffing of nurses, will be documented.

The patient’s preoperative cognitive status will be measured using neuropsychological screening, including the Montreal Cognitive Assessment (MoCA), the Trail Making Test A, and the Trail Making Test B (TMTA, TMTB). The MoCA is a 30-point brief cognitive screening tool with the following subscales: short-term memory (5 points), visuospatial ability (5 points), executive function (4 points), attention, concentration, and working memory (5 points), language (5 points), and orientation to time and place (6 points). The MoCA has a high sensitivity (0.90) and specificity (0.87) to detect patients with mild cognitive impairment [40]. The TMT is a widely used neuropsychological instrument to assess the speed of cognitive processing, visuomotor tracking, divided attention, and cognitive flexibility [34, 41–43]. The test consists of two parts (A and B). In part A, patients are instructed to draw lines connecting consecutively numbered circles from 1 to 25 in ascending order. In part B, the circles include both numbers (1–13) and letters (A-L), and the patients draw lines to connect the circles in an ascending pattern, but with the added task of alternating between the numbers and letters. Total time will be recorded in seconds. In elderly volunteers, part B had a specificity of 0.89, and a sensitivity of 0.63 for cognitive dysfunction, and 0.72 for dementia [34, 41–43].

Finally, blood samples will be measured before surgery to address the hypotheses and predictive values of serum biomakers for delirium, cognitive decline, and early-onset dementia. This examination section will take approximately 60 minutes.

### Postoperative delirium screening (t1) and postoperative laboratory measurements (t2)

All study patients will be assessed twice a day using the Confusion Assessment Method (CAM) in the ICU (CAM-ICU) or the I-CAM in the intermediate care unit (IMC). The CAM is a standardized evidenced-based tool for delirium screening at the bedside. The CAM diagnostic algorithm is based on four features of delirium: (1) acute onset or symptom fluctuation, (2) inattention, (3) disorganized thinking, and (4) altered level of consciousness. The I-CAM has a high sensitivity of 0.77 in a cohort of geriatric patients with a high prevalence of dementia and a specificity of 0.96–1.00 with inter-rater reliability of 0.95. The CAM-ICU is an adaptation of the I-CAM for critically ill patients on or off the ventilator. Initially, delirium in the ICU will be assessed by the Richmond Agitation-Sedation Scale (RASS). Afterwards, the four features of the I-CAM will be completed. The CAM-ICU shows a high sensitivity of 0.95–1.00 and a specificity of 0.89–0.93 with inter-rater reliability ranging from 0.88 to 1.0 [44–47]. Since POD symptoms often occur at night, chart reviews [48] (Additional file 4: Supplement 4) will be assessed for the first five postoperative days. This section of the investigation will take approximately 10–15 minutes per survey. Blood will be sampled again on the 7th postoperative day (t2). Finally, a surgery protocol using the data from cardiac surgery and anesthesiology will be collected using the following data: type of cardiac surgery, time of surgery (cut-to-suture-time), aortic cross-clamp time, intraoperative complications, and use/time of the heart-lung machine.

### Follow-up visit 12 months (t3) and 24 months (t4) after cardiac surgery

The follow-up visits will be performed 12 and 24 months after cardiac surgery. All participants will be invited to an in-hospital visit to be evaluated for new-onset cardiovascular, neurological, or psychiatric disorders, hospitalizations post-surgery (number and reasons of hospitalizations), COVID-19 infection and vaccination status, geriatric check (mobility, statutory level of independency, cognition, psychological symptoms, and previous hospital stay), and to undergo a short 6-item cognitive screening. The patient's current medications, the self-reported SMI, alcohol use, smoking status, and psychiatric symptoms such as hallucinations, tremor, or rigor will also be noted. Furthermore, frailty will be assessed using the CSHA Frailty Scale [38]. Postoperative cognitive impairment or early dementia status will be measured using the MoCA [40], TMTA, and TMTB [34, 41–43].

Furthermore, other neuropsychiatric symptoms will be measured using the following established and standardized instruments (Table 3): Informant Questionnaire on Cognitive Decline in the Elderly (IQCODE) [49–51], Bayer Activities of Daily Living Scale (B-ADL) [52, 53], Movement Disorder Society-Unified Parkinson's Disease Rating Scale (MDS-UPDRS) [54, 55], REM Sleep Behavior Disorder Screening Questionnaire (RBDSQ) [56], and Neuropsychiatric Inventory (NPI) [57].

**Table 3:** Neuropsychiatric assessments for follow up visits 12 and 24 months post-surgery

Questionnaire	Description	References
Informant questionnaire on cognitive decline in the elderly (IQCODE)	This shortened 7-item IQCODE is based on a structured interview in which responses of informants (e.g., spouses or relatives of patients) who know the patients well are collected. The IQCODE asks a series of questions about how the patient’s cognition and functioning have changed. Each question is scored from 1 (much improved) to 5 (much worse). For the shortened 7-item IQCODE, a cut-off point (average score) of 3.29 or higher indicates a cognitive dysfunction	[49–51]
Bayer activities of daily living scale (B-ADL)	25-item, informant-rated questionnaire was developed to assess functional disabilities in cognitively impaired elderly. The informant rates a participant´s ability to perform an activity on a scale of 1 (participant has never difficulty) to 10 (participant always has difficulty) for each of the 25 items. The total scores of the B-ADL range between values 1.00 and 10.00	[52, 53]
Movement disorder society -unified parkinson's disease rating Scale (MDS-UPDRS)	A comprehensive scale composed of four parts: Part I – Non-motor experiences of daily living (13 items); Part II Motor experiences of daily living (13 items); Part III Motor examination (18 items); Part IV – Motor complications (6 items). Each item scores from 0 to 4	[54, 55]
REM sleep behavior disorder screening questionnaire (RBDSQ)	10-item screening tool for assessment of idiopathic REM sleep behavior disorder with short questions that have to be answered be either “yes” or “no”	[56]
Neuropsychiatric inventory (NPI)	Comprehensive assessment of psychopathology in dementia measuring 10 behavioral disturbances: delusions, hallucinations, dysphoria, anxiety, agitation/aggression, euphoria, disinhibition, irritability/lability, apathy, and aberrant motor activity. Frequency and severity of each behavior are determined	[57]

Finally, blood samples will be measured to address the hypotheses and predictive values of serum biomarkers for cognitive decline and early-onset dementia. This examination section will take approximately 75 minutes.

If study participants decline an in-hospital study visit (e.g., due to concerns related to COVID-19 infection), the following instruments will be completed over the phone: MoCA [40], IQCODE [49–51], B-ADL [52, 53], MDS-UPDRS [54, 55], RBDSQ [56], and NPI [57]. In this case, we will not be able to measure a blood sample. The phone follow-up visits will take approximately 45 minutes.

### Blood sampling and biomarker measurements

Blood sampling and biomarker analysis are some of the main goals and hypotheses of this study. Up to 50 ml of blood per study visit (t0, t2, t3, t4) will be sampled. Blood collection (serum, plasma, RNA out of PAXgene tubes), processing, and storage will be performed according to local established standard operating procedures (SOPs). Serum and plasma will be briefly kept at 4 °C until processing. Serum will be stored for 45 minutes for coagulation. Serum and EDTA-plasma will be centrifugated for 10 minutes at 2,000 x g and stored as 500µl aliquots at − 80 °C. The cellular pellet from the EDTA-tube will be reconstituted in PBS and stored at –80 °C for DNA extraction. The PAXgene tube will be stored for 120 minutes at room temperature, followed by 24 hours at − 20 °C and long-term storage at − 80 °C until use.

The investigation is initially designed as a prospective single sample survey and longitudinal data collection. With the help of various protein-analytical or other measurement methods, new biomarkers for POD, cognitive decline, and dementia will be identified and validated. In addition to proteomic or metabolomic markers, genomic and epigenetic markers, such as the ApoE genotype, will be examined. The participants are therefore explicitly advised that a genetic examination of the donated material may be carried out. To protect the participants, no feedback is given about individually achieved markers, in particular predictive markers. To test our hypothesis that pre-surgical AD pathology is a risk factor to develop POD followed by cognitive decline, we will measure plasma Aβ using our recently developed two-step immunoassay [58, 59], p-tau181 [60, 61], NfL [62, 63], and GFAP [26] using SIMOA technology [64, 65].

Samples will be handled according to our recently published SOP suggestions to minimize pre-analytical effects on plasma Aβ [66]. As POD and cognitive decline are often considered to be associated with a concomitant increase in inflammatory cytokines, we want to identify markers that correlate with delirium and neurodegenerative markers. Therefore, we will also analyze a cytokine panel, including IL-1ra-, IL-6, IL-8, IL-10, and tumor necrosis factor (TNF) alpha [23, 67–69]. Furthermore, to overall understand the impact of specific cardiovascular alteration and to test the hypothesis that changes in cardiovascular markers are associated with delirium, we will analyze the cardiovascular marker growth differentiation factor 15 (GDF-15) [70].

### Study sample

In order to plan a subsequent confirmatory study, it is necessary to estimate the necessary parameters with sufficient accuracy. The sample size planning is specifically based on estimating the area under the curve (AUC) of the delirium risk assessment for the occurrence of delirium with a 95% confidence interval so that the 95% confidence interval has a width of approximately 0.05 points. It is assumed that the prevalence of POD is 50% and that there is a true AUC of 0.7. If 416 patients are analyzed, the 95% confidence interval extends approximately 0.025 points from the estimate. To compensate for possible dropouts (assumed dropout rate approx. 20%), 500 patients will be recruited. The calculation was done in nQuery 8.

### Statistical analysis

Prognostic quality measures are calculated in order to determine the prognostic properties of continuous endpoints and delirium risk assessment scores in predicting the development of POD, postoperative cognitive decline, and AD. Specifically, a receiver operating characteristics (ROC) analyses is conducted for each of the outcomes and the associated area under the curve (AUC) is reported with optimal cut-off points (simultaneous maximization of sensitivity and specificity, as well as according to Youden). Furthermore, sensitivity, specificity, positive and negative predictive values ​​for the optimal cut-off point are reported. The observed incidence risk factors for the development of POD, cognitive decline, AD, or Lewy body dementia will be investigated using logistic regression or, in the case of time-to-event data, cox regression. Effect sizes are reported as odds ratio or hazard ratio. As far as possible, all key figures are given with a 95% confidence interval. In addition to the univariate consideration of the individual parameters, a multivariate consideration of predictive factors is also planned. For this purpose, machine learning methods for supervised learning are employed (e.g. multivariate logistic regression) and candidate models are trained on a training data set (70% of the data collected). Different models and their corresponding accuracy are compared using a test data set (30% of the data collected). The division of data into test and training data set is random.

### Data management

All outcomes are entered into a Good Clinical Practice (GCP) compliant database (SecuTrial^®^), configured for this trial. The configuration includes univariate checks for plausibility, such as range checks. Data are regularly reviewed for completeness by qualified personnel and locked after review. A blinded data review (without knowledge of the development of delirium) to assess data quality is performed prior to database lock. After database lock, the data set is archived for at least 10 years on a digital medium within the trial master file. An anonymized copy of the data set is provided alongside the publication to ensure the reproducibility of results. The investigators follow the Findability, Accessibility, Interoperability, and Reuse (FAIR) Guiding Principles for scientific data management and stewardship [71].

## Discussion

Postoperative delirium is a common complication of cardiac surgery associated with prolonged hospitalization, postoperative complications, and increased risks of cognitive decline, dementia, and mortality [10–12]. Geriatric patients, patients after cardiac surgery, and intensive care patients are at a high risk of developing POD [3–7, 9]. Additionally, previous research shows an increase in neurodegenerative and neuroinflammatory markers after cardiac surgery [14, 16]. The ability to identify moderate- to high-risk patients prior to surgery is essential, as these individuals might be targeted for delirium prevention programs and more rigorous screening following surgery to identify POD. Delirium prevention and early identification/treatment has the potential to significantly improve in-hospital and post-discharge outcomes, including postoperative complications, length of stay, and mortality [31]. While there are well-established predictors for delirium incidence, an established standard assessment or measurement of a biomarker for risk of POD and its severity is not available [33, 35, 36]. Further research is needed to establish a reliable screening instrument and measurements of biomarkers for the risk of POD, cognitive decline, or dementia in patients undergoing cardiac surgery. Early identification of patients at high risk for delirium and cognitive decline can potentially help to implement specific non-pharmacological interventions for delirium prevention [34, 72].

FINDERI is a prospective, single-center, observational study. In total, 500 patients aged over 50 undergoing cardiac surgery will be recruited at the Department of Cardiovascular and Thoracic Surgery of the University Medical Center Göttingen, Germany. Data collection will extends from the time of admission (t0; preoperative screening), through t1 on the 1st–5th postoperative days (delirium screening), t2 on the 7th postoperative day (laboratory measurements), t3 after 12 months postoperatively, and t4 after 24 months postoperatively.

The primary aim of our study is to analyze the reliability, sensitivity, and specificity of an existing delirium risk assessment in context of cardiac surgery. Secondary aims are i) to identify specific preoperative risk factors and perioperative exposition factors for POD, cognitive decline, and accelerated dementia after cardiac surgery, and ii) to identify serum biomarkers that predict the incidence of POD, cognitive decline, or dementia in the context of cardiac surgery.

Our FINDERI study design has several strengths. The longitudinal study design with two year follow up has the potential to identify patients with cognitive dysfunction or accelerated dementia after cardiac surgery. In addition, the study utilizes the highest standards for delirium detection, including the CAM, CAM-ICU, and chart review. Finally, the study analyzes a broad and representative population in cardiac surgery. However, this study has several limitations. Firstly, we will focus our primary and secondary hypotheses on identifying pre- and perioperative delirium risk factors, and not on the impact of delirium on morbidity, mortality, and hospital utilization of patients undergoing cardiac surgery or its treatment. Secondly, if study participants decline an in-hospital follow up study visit (e.g., due to a high risk of COVID-19 infection), we will not be able to measure a blood sample. Third, further research will be needed to replicate our findings in a larger multicenter, prospective, observational study.

From the clinical perspective, this study might help to identify patients at high risk for delirium, and to develop interventions to prevent delirium prior to cardiac surgery. Furthermore, this study might help to identify important biological mechanisms by which surgery is associated with delirium. Finally, the identification of specific blood biomarkers might help to predict delirium, cognitive decline, and dementia in patients undergoing cardiac surgery.

## Supplementary Information

**Additional file 1.** Delirium risk assessment

**Additional file 2.** Medical history, cardiovascular risk factors, medication, and sociodemographic data

**Additional file 3.** Outcome-oriented Nursing Assessment (ePA-AC)

**Additional file 4.** Chart review for identification of a postoperative delirium on Day 1-5

## Data Availability

Research data will be stored and managed at the University of Göttingen Medical Center in Germany. The anonymized original data set will be published in a certified data repository for future use after completion of the trial. The principal investigators will form a subgroup for approving requests from external researchers to access the data. The principal investigators follow authorship eligibility guidelines, and designed a publication strategy prior to study initiation. The investigators follow the FAIR Principles for scientific data management and stewardship.

## Abbreviations

Aβ_42_: Amyloid-β_42_
ACS: American College of Surgeons
AD: Alzheimer’s disease
ASA: American Society of Anesthesiologists
AUC: Area under the curve
B-ADL: Bayer activities of daily living scale
CAM: Confusion assessment method
CCL2: CC-chemokine ligand 2
CRP: C-reactive protein
CSHA: Canadian study of health and aging
CSF: Cerebrospinal fluid
DRKS: German clinical trials register
ePA-AC: AcuteCare 1.1
FAIR: Findability, accessibility, interoperability, and reuse
FGF: Fibroblast growth factor
GCP: Good clinical practice
GFAP: Glial fibrillary acidic protein
GDF: Growth differentiation factor
HR: Hazard ratio
ICU: Intensive care unit
IL-6: Interleukin-6
IMC: Intermediate care unit
IQCODE: Informant questionnaire on cognitive decline in the elderly
MDS-UPDRS: Movement disorder society-unified Parkinson's disease rating scale
MoCA: Montreal cognitive assessment
NfL: Neurofilament light chain
NPI: Neuropsychiatric inventory
NSQIP-SC: National surgical quality improvement program risk calculation for serious complications
OR: Odd ratio
POD: Postoperative delirium
RASS: Richmond agitation-sedation scale
RBDSQ: REM sleep behavior disorder screening questionnaire
ROC: Receiver operating characteristics
SMI: Subjective memory impairment
SOP: Standard operating procedures
TMTA: Trail making test A
TMTB: Trail making test B
TNF: Tumor necrosis factor
