# Supplementary material for: Identification of risk factors for delirium, cognitive decline, and dementia after cardiac surgery (FINDERI—find delirium risk factors): a study protocol of a prospective observational study
Source: BMC Cardiovasc Disord. 2022 Jun 30;22:299. doi: 10.1186/s12872-022-02732-4 (PMC9245863; doi:10.1186/s12872-022-02732-4)
Supplement: Supplementary file 1 — Additional file 1. Delirium risk assessment [file 12872_2022_2732_MOESM1_ESM.docx]

**Supplementary File S1.** Delirium risk assessment

DELIRIUM RISK ASSESSMENT
(modified from PAWEL trial)

Identification of Risk Factors for Delirium, Cognitive Decline, and Dementia after Cardiac Surgery - FINDERI (Find Delirium Risk Factors)

**A prospective single-center observational study**

**SecuTrial-ID :**

**Date:**

| **Impairment preceding the current event/ illness** | | | |
| --- | --- | --- | --- |
| **Mobility** | - Gait insecurity and/or - Repeated falls and/ or - Mobility aid/ wheelchair | - yes | - no |
| **Independence** | - Help with bathing/ getting dressed and/or - Incontinence and/ or - Malnutrition | - yes | - no |
| **Cognition** | - Memory impairment and/ or - Disorientation/ Confusion | - yes | - no |
| **Psyche** | - Persistent dejection and/ or - Persistent lethargy | - yes | - no |
| **Hospital stays** | ≥ 2 hospital stays within the last 12  months | - yes | - no |
|  | Number of yes-answers(sum) |  |  |
| **≥ 2 yes-answers:** | | **Likely to be a geriatric patient ☐** | |

**6-item Cognitive Impairment Test (6CIT)**

|  | | Points: |
| --- | --- | --- |
| What year is it? | Correct: 0 points  Incorrect: 4 points |  |
| What month is it? | Correct: 0 points  Incorrect: 3 points |  |
| Please remember the following address: Erika Meyer, Wacholderweg 17, Sindelfingen | | |
| About what time is it (within one hour)? | Correct: 0 points  Incorrect: 3 points |  |
| Please count backwards from 20 to one. | Correct: 0 points  1 error: 2 points  > 1 error: 4 points |  |
| Please repeat the address: | Correct: 0 points |  |
| Erika Meyer, Wacholderweg 17, Sindelfingen | 1 error: 2 points |  |
|  | 2 error: 4 points |  |
|  | 3 error: 6 points |  |
|  | 4 error: 8 points |  |
|  | All wrong: 10 points |  |
| Total points: | |  |
| 0-7 = normal | |  |
| 8-9 = mild cognitive impairment | | ☐ |
| 10-28 = considerable cognitive impairment | | ☐☐ |

| **General information** | | |
| --- | --- | --- |
|  | | |
| Likely to be a geriatric patient according to geriatric check | Yes | ☐ |
| 6CIT test result:  Mild cognitive impairment Considerable cognitive impairment | Yes Yes | ☐  ☐☐^1^ |
| Age >80 | Yes | ☐ |
| Lab data: | | |
| Increased creatinine levels | Yes | ☐ |
| Increased CRP levels | Yes | ☐ |
| Reduced Hb | Yes | ☐ |
| Electrolytes out of norm | Yes | ☐ |
| Reduced protein levels | Yes | ☐ |
| ASA ≥ 3 | Yes | ☐ |
| More than 6 medications per day | Yes | ☐ |
| Medication with potential to cause delirium (see list; at least 1 of those) | Yes | ☐ |
| Dementia diagnosis | Yes | ☐ |
| Depression diagnosis | Yes | ☐ |
| Stroke diagnosis | Yes | ☐ |
| Parkinson’s diagnosis | Yes | ☐ |
| Level of Care (German care level ≥ 1) | Yes | ☐ |
| Nursing home resident | Yes | ☐ |
| Did you suffer from delirium/ acute confusion during another hospital stay? | Yes | ☐ |
| Did you trip or fall/ nearly fall during the last few months? | Yes | ☐ |
| How many/Which alcoholic beverages do you consume per day on average? Wine/ beer/ other: | More than 5 | ☐ |
| Current smoking behaviour: Daily | Correct | ☐ |
| Do you feel like your memory is declining? If so, does that significantly bother you? | Yes  Yes, it bothers me significantly | ☐  ☐☐^1^ |
| Manual force:  < 20 kg for women  < 32 kg for men | Yes | ☐ |
| **Sum of checked items:** | |  |

**Evaluation (Sum of checked items):**

< 2: no further measures necessary; 2-4: Further clarification concerning the risk for delirium ➐ if necessary, consult geriatrician/ neurologist; > 4: Note down and apply measures for further actions, suitable measures can also be found in the therapeutic concept. If existing, SOP’s (Standard Operating Procedures) should be applied to guarantee an optimal support for patients at risk during their in-patient stay.

1 Counts as two checked items!
