# Supplementary material for: Identification of risk factors for delirium, cognitive decline, and dementia after cardiac surgery (FINDERI—find delirium risk factors): a study protocol of a prospective observational study
Source: BMC Cardiovasc Disord. 2022 Jun 30;22:299. doi: 10.1186/s12872-022-02732-4 (PMC9245863; doi:10.1186/s12872-022-02732-4)
Supplement: Supplementary file 2 — Additional file 2. Medical history, cardiovascular risk factors, medication, and sociodemographic data [file 12872_2022_2732_MOESM2_ESM.docx]

**Supplementary File S2.** Medical history, cardiovascular risk factors, medication, and sociodemographic data

# MEDICAL HISTORY AND CARDIOVASCULAR RISK FACTORS

Identification of Risk Factors for Delirium, Cognitive Decline, and Dementia after Cardiac Surgery - FINDERI (Find Delirium Risk Factors)

**A prospective single-center observational study**

| **Date:** |
| --- |
| **SecuTrial-ID:** |

| **Month of birth:** | **Year of Birth:** |
| --- | --- |
| **Body weight in kg:** | **Body height in cm:** |
| **Gender:**   - male 🞆 female | |

### PRE-EXISTING CONDITIONS

**Coronary heart disease** 🞆 yes 🞆no 🞆unknown

### If yes:

- 1VD 🞆 2VD 🞆 3VD Cardiac catheter examination, when? yyyy

**Heart attack:** 🞆 yes 🞆no 🞆unknown If yes:

When (most recent) yyyy

**Interventional coronary revascularisation:** 🞆yes 🞆no 🞆unknown If yes:

When (most recent)? yyyy

**Coronary bypass surgery:** 🞆yes 🞆no 🞆unknown If yes:

When (most recent)? yyyy

**Heart failure** 🞆 yes 🞆no 🞆unknown If yes:

NYHA-class 🞆 I 🞆 II 🞆III 🞆 IV

**Implanted heart pacemaker or defibrillator?** 🞆no 🞆Heart pacemaker 🞆defibrillator If yes:

When (most recent)? yyyy

**Heart valve disease** 🞆 yes 🞆no 🞆unknown

### If yes:

- Aortic valve disease 🞆 stenosis 🞆 insufficiency 🞆 combined
- Classification 🞆 mild 🞆 moderate 🞆 severe
- Mitral valve disease 🞆 stenosis 🞆 insufficiency 🞆 combined
- Classification 🞆 mild 🞆 moderate 🞆 severe
- Tricuspid valve disease 🞆 stenosis 🞆 insufficiency 🞆 combined
- Classification 🞆 mild 🞆 moderate 🞆 severe

| **Aortic aneurysm** | - yes | - no | - unknown |
| --- | --- | --- | --- |
| **Aortic dissection** | - yes | - no | - unknown |
| **Carotid artery stenosis** | - yes | - no | - unknown |
| **Atrial fibrillation/flutter** | - yes | - no | - unknown |
| **Endocarditis** | - yes | - no | - unknown |

**Other cardiac diseases** (cardiomyopathy, history of myocarditis)

Please indicate;

Please indicate;

Please indicate;

| **PAD** | - yes | - no | - unknown |
| --- | --- | --- | --- |
| **Stroke/TIA** | - yes | - no | - unknown |
| **Renal insufficiency** | - yes | - no | - unknown |
| **Currently requiring dialysis** | - yes | - no | - unknown |
| **Tumor disease (within the last 5 years)** | - yes | - no | - unknown |
| **Thyroid disease** | - yes | - no | - unknown |
| If yes, what kind of disease: |  |  |  |

| **COPD** | - yes | - no | - unknown |
| --- | --- | --- | --- |
| **Diabetes mellitus** | - yes | - no | - unknown |
| **Depression** | - yes | - no | - unknown |
| **Anxiety disorder** | - yes | - no | - unknown |
| **Alcohol/drug addiction** | - yes | - no | - unknown |
| **Dementia** | - yes | - no | - unknown |
| **Parkinson’s disease** | - yes | - no | - unknown |
| **Subjective evaluation of memory** | |  |  |
| **Do you feel like your memory is declining?**   - yes - no - don’t know (no information) | |  |  |
| **If yes, does that bother you?**   - no - yes, it bothers me. - yes, it bothers me significantly. - don’t know; no information - not correct | |  |  |

**Hearing aids /glasses**

| **Hearing aids** | - no | - yes |
| --- | --- | --- |
| **Glasses** | - no | - yes |

| **Current smoking behaviour?**   - daily, cigarettes, cigars, or pipes per day - less than daily, cigarettes or pipes per week - never |
| --- |
| **If you are not a current smoker, did you use to smoke?**   - daily, cigarettes, cigars, or pipes per day - less than daily, cigarettes or pipes per week - never |
| **If former smoker:**  **How many years/ months ago did you stop smoking?**  years months |
| **How often do you drink alcoholic beverages?**  **Beer/wine:** ☐ never   - less than once a month - 1-3 times a month - 1-4 times a week - 5-6 times a week - daily   **Spirits** ☐ never   - less than once a month - 1-3 times a month - 1-4 times a week - 5-6 times a week - daily |
| **How often do you drink 3 or more alcoholic beverages at the same time?**   - never ☐ 1-4 times a week - less than once a month ☐ 5-6 times a week - 1-3 times a month ☐ daily |

**Cardiovascular Medication (Current medication)**

| **ACE-inhibitors** (e.g., ramipril) | - yes | - no | - unknown |
| --- | --- | --- | --- |
| **AT1-receptor antagonists** (e.g., valsartan) | - yes | - no | - unknown |
| **Aldosterone antagonists** (e.g., spironolactone) | - yes | - no | - unknown |
| **Sacubitril/valsartan** (Entresto®) | - yes | - no | - unknown |
| **Amiodarone** (e.g., Cordarex®) | - yes | - no | - unknown |
| **Other antiarrhythmics** | - yes | - no | - unknown |
| **Beta blockers** (e.g., bisoprolol) | - yes | - no | - unknown |
| **Calcium channel antagonists** (e.g., amlodipine) | - yes | - no | - unknown |
| **Thiazide diuretics** (e.g., HCT) | - yes | - no | - unknown |
| **Loop diuretics** (e.g., torsemide) | - yes | - no | - unknown |
| **Cardiac glycosides** (e.g., digoxin) | - yes | - no | - unknown |
| **Nitrate** | - yes | - no | - unknown |
| **Ranolazine** (e.g., Ranexa®) | - yes | - no | - unknown |
| **Ivabradine** (e.g., Procoralan®) | - yes | - no | - unknown |
| **Statin** (e.g., Atorvastatin) | - yes | - no | - unknown |
| **Other lipid reducers** | - yes | - no | - unknown |

| **Anticoagulants (Current medication)** |  |  |  |  |  |  |
| --- | --- | --- | --- | --- | --- | --- |
| **Aspirin** | | | | - yes | - no | - unknown |
| **Thienopyridine**  (e.g., clopidogrel, prasugrel, ticagrelor) | | | | - yes | - no | - unknown |
| **Vitamin K antagonist**  (e.g., phenprocoumon) | | | | - yes | - no | - unknown |
| **Direct oral anticoagulant**  (e.g., apixaban, edoxaban, dabigatran) | | | | - yes | - no | - unknown |

**Antidiabetics (Current medication)**

| **Insulin** | - yes | - no | - unknown |
| --- | --- | --- | --- |
| **Oral antidiabetic** (e.g., metformin) | - yes | - no | - unknown |

**Analgesics (Current medication)**

| **NSAID** (e.g., ibuprofen) | - yes | - no | - unknown |
| --- | --- | --- | --- |
| **Opioids** (e.g., tramadol, oxycodone) | - yes | - no | - unknown |

**Hormones (Current medication)**

| **Thyroid medicine** | - yes | - no | - unknown |
| --- | --- | --- | --- |
| **Oral contraceptive** | - yes | - no | - unknown |
| **Other hormone preparations** | - yes | - no | - unknown |
| **Psychotropic drugs (Current medication)** |  |  |  |
| **Antidepressants/anxiolytics** |  |  |  |
| **SSRI** (e.g., sertraline) | - yes | - no | - unknown |
| **SNRI** (e.g., venlafaxine) | - yes | - no | - unknown |
| **Tricyclic antidepressants** (e.g., amitriptyline) | - yes | - no | - unknown |
| **Tetracyclics** (e.g., mirtazapine) | - yes | - no | - unknown |
| **MAO inhibitor** (e.g., tranylcypromine) | - yes | - no | - unknown |
| **Agomelatine** (e.g., Valdoxan®) | - yes | - no | - unknown |
| **Others,** which one? | - yes | - no | - unknown |
| **Sleeping medication/sedatives** |  |  |  |
| **Benzodiazepines** | - yes | - no | - unknown |
| **Zolpidem/Zopiclone** | - yes | - no | - unknown |

**Others,** which one? 🞆 yes 🞆no 🞆unknown

### Antipsychotic/neuroleptic

**Phenothiazines** (e.g., promethazine, levomepromazine) 🞆 yes 🞆 no 🞆 unknown **Butyrophenone** (e.g., pipamperone, melperone, haloperidol) 🞆 yes 🞆 no 🞆 unknown **Atypical antipsychotics** (e.g., quetiapine, risperidone) 🞆 yes 🞆 no 🞆 unknown
**Other,** which one? 🞆 yes 🞆 no 🞆 unknown

**Other medication (not mentioned above)**

**PPI** (e.g., pantoprazole) 🞆 yes 🞆 no 🞆 unknown Please name

Please name

Please name

Please name

Please name

Please name

# SOCIODEMOGRAPHIC DATA

## Identification of patients at risk of developing delirium and a delayed and prolonged cognitive decline after heart surgery - FINDERI (Find delirium risk factors)

**A prospective single-center observational study**

| **Date:** |
| --- |
| **SecuTrial-ID:** |

| **1.** | **Please state your marital status.**  living with spouse/ partner   - married, living separately from spouse - single - divorced - widowed |
| --- | --- |
| **2.** | **Do you have children?**   - no - yes, number: |
| **3.** | **Migrant background: Did you, your parents, or your grandparents grow up in a country different from the one you are currently residing in?**   - no - yes, 🞆 yourself 🞆 parents 🞆 grandparents   Native language: |

| **4.** | **What is your highest level of education?**   - No secondary school or elementary schooling - Secondary school - Intermediate modern secondary school - Graduated from the polytechnic secondary school Year 11 (before 1965: Year 9) - Advanced technical college certificate - General higher education entrance qualification (Abitur) or subject-related entrance qualification - Other school-leaving qualification: |
| --- | --- |
| **5**. | **Did you complete a vocational training?**   - no - yes, 🡪 ☐ Apprenticeship ☐ Study |
| **6.** | **Which profession did you mainly pursue?**  Mainly pursued profession: Duration of years working: years  Pension age: |
| **7**. | **With whom do you live at home? (multiple answers are accepted)**   - alone ☐ flat-sharing community - child/ children ☐ nursing/Assisted living facility - spouse/ partner - other relatives |
| **8.** | **Do you posses a care degree? If so, which one?**   - No - Yes Care degree:   **Did you apply for a care degree?**   - Yes - No |
