# Supplementary material for: Identification of risk factors for delirium, cognitive decline, and dementia after cardiac surgery (FINDERI—find delirium risk factors): a study protocol of a prospective observational study
Source: BMC Cardiovasc Disord. 2022 Jun 30;22:299. doi: 10.1186/s12872-022-02732-4 (PMC9245863; doi:10.1186/s12872-022-02732-4)
Supplement: Supplementary file 3 — Additional file 3. Outcome-oriented Nursing Assessment (ePA-AC) [file 12872_2022_2732_MOESM3_ESM.docx]

**Supplementary File S3.** Outcome-oriented Nursing Assessment (ePA-AC)

1. **Was the EpAC raised by nursing within the last 24h:**

- Yes
- No:
  - Care was understaffed in the last 24h
  - Nursing has been replaced by a substitute or agency worker who does not routinely work on the ward
  - Standard is not established
  - Nursing needs training on ePA-AC
  - Further reasons

____________________________________________________________

1. **master data:**
   1. Age years

2.2 Aids (locomotion/mobility):

- none
- needs
  - Which? _________________

1. **Mobility:**
   1. SPF locomotion:

- 1: no ability/bed rest

| - 2: severely impaired |  |
| --- | --- |
| - 3: low impairment |  |
| - 4: full ability |  |

- 1. History of falls:
- Yes
- No
- Not evaluated
  1. Current fall event:
- Yes
- No

1. **Body care and dressing:**
   1. SPF Body care upper body:

- 1: no ability/bed rest

| - 2: severely impaired |  |
| --- | --- |
| - 3: low impairment |  |
| - 4: full ability |  |

- 1. SPF Body care lower body:
- 1: no ability/bed rest

| - 2: severely impaired |  |
| --- | --- |
| - 3: low impairment |  |
| - 4: full ability |  |

- 1. SPF Dressing / undressing upper body:

| - 1: no ability/bed rest  \| - 2: severely impaired \|  \| \| --- \| --- \| \| - 3: low impairment \|  \| \| - 4: full ability \|  \|  - 1. SPF Dressing / undressing lower body: - 1: no ability/bed rest  \| - 2: severely impaired \|  \| \| --- \| --- \| \| - 3: low impairment \|  \| \| - 4: full ability \|  \| |  |
| --- | --- | --- | --- | --- | --- | --- | --- | --- | --- | --- | --- | --- | --- |

1. **Nutrition:**
   1. SPF Eating:

- 1: no ability/bed rest

| - 2: severely impaired |  |
| --- | --- |
| - 3: low impairment |  |
| - 4: full ability |  |

1. **Excretion:**
   1. SPF Urine excretion:

- 1: no ability/bed rest

| - 2: severely impaired |  |
| --- | --- |
| - 3: low impairment |  |
| - 4: full ability |  |

- 1. SPF Stool excretion:
- 1: no ability/bed rest

| - 2: severely impaired |  |
| --- | --- |
| - 3: low impairment |  |
| - 4: full ability |  |

1. **Cognition and Awareness:**
   1. Consciousness/vigilance:

- 1: comatose
- 2: soporific
- 3: somnolent
- 4: awake
  1. Orientation (person, place, time, situation)
- 1: to no qualities

| - 2: to one or two qualities |  |
| --- | --- |
| - 3: to three qualities |  |
| - 4: to all qualities |  |

- 1. Acquire knowledge:
- 1: no ability/bed rest

| - 2: severely impaired |  |
| --- | --- |
| - 3: low impairment |  |
| - 4: full ability |  |

- 1. Daily life skills:

| - 1: no ability/bed rest  \| - 2: severely impaired \|  \| \| --- \| --- \| \| - 3: low impairment \|  \| \| - 4: full ability - Not evaluated \|  \| |  |
| --- | --- | --- | --- | --- | --- | --- | --- |

- 1. Attention:
- 1: continuously impaired

| - 2: temporarily impaired |  |
| --- | --- |
| - 3: Not impaired |  |
| - 4: Not evaluated   1. Fall/delir risk-increasing medications: - 1: Yes  \| - 2: No \|  \| \| --- \| --- \| |  |

| \| \|  \| \| --- \| \|  \| \|  \| \| --- \| \| \| --- \| --- \| --- \| --- \| --- \| |
| --- | --- | --- | --- | --- | --- |
| 1. **Communication and interaction:**    1. Self-initiated activities:  - 1: No self-initiated activities  \| - 2: few self-initiated activities \|  \| \| --- \| --- \| \| - 3: Increased self-initiated activities \|  \| \| - 4: Initiates own activities comprehensively \|  \|  - 1. Characteristics of challenging behavior: - 1: Agitates non-aggressive behavior (physical/verbal)  \| - 2: agitates aggressive behavior (physical/verbal) \|  \| \| --- \| --- \| \| - 3: passive/ apathetic behavior \|  \| \| - 4: none \|  \| |
| 1. **Sleep:**     1. sleep and wake rhythm:  - 1: impaired  \| - 2: no impairment \|  \| \| --- \| --- \| \| - 3: Not evaluated  1. **Risk:** \|  \|   **10.1**. Confusion, dementia, delirium   - 1: Yes  \| - 2: No \|  \| \| --- \| --- \| \|  \|  \|   **_____________________________________________________________** |
|  |
|  |

|  |
| --- |

| \| \|  \| \| --- \| \| \| **Required items: 21/71 of the documented** \| \| --- \| \|  \| \| \|  \| \| --- \| \| \| \| --- \| --- \| --- \| --- \| --- \| --- \| --- \| |
| --- | --- | --- | --- | --- | --- | --- | --- |
