# Supplementary material for: Identification of risk factors for delirium, cognitive decline, and dementia after cardiac surgery (FINDERI—find delirium risk factors): a study protocol of a prospective observational study
Source: BMC Cardiovasc Disord. 2022 Jun 30;22:299. doi: 10.1186/s12872-022-02732-4 (PMC9245863; doi:10.1186/s12872-022-02732-4)
Supplement: Supplementary file 4 — Additional file 4. Chart review for identification of a postoperative delirium on Day 1-5 [file 12872_2022_2732_MOESM4_ESM.docx]

**Supplementary File S4.** Chart review for identification of a postoperative delirium on day 1-5

| 1. **Consciousness and attention impairment**   (“agitated,” “sleepy;” scales: RASS, GCS, reaction to pain stimulation, ability to make conversation, physical reaction to stimuli, eyes open as impulse response, lethargic, racing thoughts, slowing down) | O yes O no |
| --- | --- |
| 1. **Apperception impairment (memory, orientation)**   (“increasingly confused,” “disoriented” in at least one of the 4 qualities; hallucinations etc.) diagnosed by personal or indirect anamnesis; psychiatric or neurological consultation | O yes O no |
| 1. **Acute and fluctuating course**   (observation at least three times a day; any variation of the mental state) | O yes O no |
| 1. **Detection of an organic cause**   (clinical observation, incl. AVPU scale, RR syst., heart rate, respiratory rate, oxygen saturation, temperature, CRP, urea, creatinine) | O yes O no |
| 1. **Psychomotor activity impairment**   (fiddling, apathetic, pulling tubes and/or removing bandages; screaming; being verbally/physically aggressive; delayed or no physical response) | O yes O no |
| 1. **Prescription of antipsychotic drugs for delirium**   (e.g., Haloperidol, Melperon, Pipamperon, Dextor, Quetiapin, Clonidin) | O yes O no |
| 1. **CAM-ICU positive** | O yes O no |
| 1. **ICAM positive** | O yes O no |
| 10.) **Delirium**  Onset: Stop: Duration:  **Criteria for defining delirium according to DSM V:**  **Item 1 + 2 + 3 = Delirium**  If the above-named items do not all apply, but item 4,5 and/or 6 are positive: **Supervision with Prof. v. Arnim** | O  yes      O  no  hypoactive O  hyperactive O  Mixed form O |
| **Clinical notes about the patient:**  Enter free text here |  |
| **Results of the supervision:**  Enter free text here | Delirium:  O yes O no |
